# Comparing gradient boosting machine and Bayesian threshold BLUP for genome‐based prediction of categorical traits in wheat breeding

**DOI:** 10.1002/tpg2.20214

**Published:** 2022-05-10

**Authors:** Osval Antonio Montesinos‐López, Henry Nicole Gonzalez, Abelardo Montesinos‐López, María Daza‐Torres, Morten Lillemo, José Cricelio Montesinos‐López, José Crossa

**Affiliations:** ^1^ Facultad de Telemática Univ. de Colima Colima Colima 28040 México; ^2^ Univ. Tecnológica de Manzanillo Manzanillo Colima México; ^3^ Centro Universitario de Ciencias Exactas e Ingenierías (CUCEI) Univ. de Guadalajara Guadalajara Jalisco 44430 México; ^4^ Dep. of Public Health Sciences Univ. of California Davis CA 95616 USA; ^5^ Dep. of Plant Sciences Norwegian Univ. of Life Sciences IHA/CIGENE, P.O. Box 5003, NO‐1432 Ås Norway; ^6^ Dep. de Estadística Centro de Investigación en Matemáticas (CIMAT) Guanajuato Guanajuato 36023 México; ^7^ Colegio de Postgraduados Montecillos Edo. de México 56230 México; ^8^ Biometrics and Statistics Unit, Genetic Resources Program International Maize and Wheat Improvement Center (CIMMYT) Km 45, Carretera México‐Veracruz 52640 México

## Abstract

Genomic selection (GS) is a predictive methodology that is changing plant breeding. Genomic selection trains a statistical machine‐learning model using available phenotypic and genotypic data with which predictions are performed for individuals that were only genotyped. For this reason, some statistical machine‐learning methods are being implemented in GS, but in order to improve the selection of new genotypes early in the prediction process, the exploration of new statistical machine‐learning algorithms must continue. In this paper, we performed a benchmarking study between the Bayesian threshold genomic best linear unbiased predictor model (TGBLUP; popular in GS) and the gradient boosting machine (GBM). This comparison was done using four real wheat (*Triticum aestivum* L.) data sets with categorical traits measured in terms of two metrics: the proportion of cases correctly classified (PCCC) and the Kappa coefficient in the testing set. Under 10 random partitions with four different sizes of testing proportions (20, 40, 60, and 80%), we compared the two algorithms and found that in three of the four data sets, the GBM outperformed the TGBLUP model in terms of both metrics (PCCC and Kappa coefficient). In the larger data sets (Data Sets 3 and 4), the gain in terms of prediction accuracy of the GBM was considerably significant. For this reason, we encourage more research using the GBM in GS to evaluate its virtues in terms of prediction performance in the context of GS.

**Abbreviations**
Bed2IR: bed planting with two irrigation levels
Bed5IR: bed planting with five irrigations
DL: deep learning
DTHD: days to heading
EHT: early heat
EYT: elite yield trial
Flat5IR: flat planting and five irrigations
FlatDrip: flat planting with drip irrigation
GBM: gradient boosting machine
GS: genomic selection
LHT: late heat
PCCC: proportion of cases correctly classified
SVM: support vector machine
TGBLUP: threshold genomic best linear unbiased predictor model

## 1 INTRODUCTION

Genomic selection (GS), the methodology proposed by Meuwissen et al. (2001), is a novel approach for predicting complex traits that exploits genetic markers and consists of developing a training population (with phenotypic and genotypic information) with which a statistical machine‐learning algorithm is trained and then used for making predictions for a testing breeding population (with only genotypic information). Finally, the selection of candidate individuals in the testing breeding population is made based on the predicted phenotypic values or breeding values. In plant breeding, GS offers ample opportunities to increase the genetic gain of complex traits per unit time and cost (Bhat et al., 2016) when it is correctly applied (Zhong et al., 2009, Heffner et al., 2010).

Genomic selection is very attractive with regard to phenotypic selection since in complex traits (yield, quality, biotic and abiotic stress, etc.) it provides (a) high selection accuracy, (b) reduced cycle time, (c) greater gain per unit time, (d) precision and accuracy, and (e) expected results (Bhat et al., 2016). In this way, GS avoids the long period (5–12 yr) needed to develop a crop cultivar based only on phenotypic selection, which is less effective for complex and low heritable traits (Tuberosa, 2012). For this reason, GS is revolutionizing plant breeding and has been implemented in many crops like wheat (*Triticum aestivum* L.), maize (*Zea mays* L.), cassava (*Manihot esculenta* Crantz), chickpea (*Cicer arietinum* L.), and rice (*Oryza sativa* L.), among others (Crossa et al., 2013, 2017; Huang et al., 2019; Meuwissen et al., 2013; Môro et al., 2019; Roorkiwal et al., 2016; Salam & Smith, 2016; Smallwood et al., 2019; Vivek et al., 2017; Wolfe et al., 2017). In this vein, many more new breeding programs are moving from conventional breeding to GS.

However, the successful implementation of GS must take several factors into consideration such as (a) selecting a representative (training) set (Guo et al., 2019), (b) guaranteeing the quality of genotypic and phenotypic data in the training set (Edwards et al., 2019), (c) having a representative sample (good coverage) of the markers in the complete genome, and (d) selecting the best statistical machine‐learning method. The selection of the optimal statistical machine‐learning method is not an easy task because of the ‘no‐free‐lunch’ theorem that states that there is no best single machine‐learning algorithm across all possible prediction problems (Wolpert, 1996; Wolpert & Macready, 2005). For this reason, the development of new statistical machine‐learning methods, as well as the adoption and exploration in GS of existing statistical machine‐learning algorithms, is an important field of research.

The two most popular linear methods used in genomic prediction are mixed models (linear models with fixed and random effects) and Bayesian methods (Bayesian ridge regression, BayesA, BayesB, BayesC, and Bayesian lasso). However, recently, many popular statistical machine‐learning models, like support vector machine (SVM) (Montesinos‐López, et al., 2019), deep learning (DL) (Montesinos‐López, et al., 2021a, 2021b), and random forest (Sarkar et al., 2015) have also been explored in the context of GS. Some of these methods have been successfully applied in many other fields since they are powerful at capturing complex nonlinear patterns like those found in the context of GS.

While machine‐learning methods focus on prediction without using a pre‐existing model, statistical approaches formalize relations between variables in the form of explicit mathematical models with parameters that are estimated. In GS, or any other research area, one approach is to build the model from theory and estimate its parameters based on the available data. However, in practical situations, models are not easy to develop, and thus, machine‐learning applications are used for building supervised nonparametric models (for regression and classification) like DL, SVM, or any other algorithm. Consequently, the model is created from the data.

As suggested by Freund and Schapire (1997), boosting is a general framework for constructing an extremely accurate prediction with various roughly accurate predictions. Undertaken by Friedman (2001) and Natekin and Knoll (2013), the gradient boosting machine (GBM) investigates how to build predictive models through back fittings and nonparametric regressions. Rather than building a single model, the GBM starts by developing an initial model and constantly fits new models through loss function minimization to produce the most precise model (Natekin & Knoll, 2013). Within machine‐learning techniques, GBM is a family of powerful methods that have shown success in several practical applications. In a specific machine‐learning task, a simple model can first be created, or an ensemble of models can be developed for some particular learning tasks. Ensembles are created by linking multiple simple models in the best possible manner to produce a complex model. In practice, the ensemble approach combines several simple models to attempt to build a stronger ensemble prediction.

Random forest and GBM are examples of machine‐learning ensemble techniques. Random forest and GBM are models that may account for non‐additive effects using fast algorithms that account for a large number of covariates and interactions and can be used in both classification and regression problems. Random forest and GBM are ensemble learning methods that make predictions (regression or classification) by combining the outputs from individual trees. They differ in the way the trees are built and the way the results are combined. A random forest is created using a process called ‘bagging’ in which each decision tree is used as a parallel estimator. Each decision tree is fit to a subsample taken from the whole data set. Decision trees in GBM are linked sequentially (i.e., in series) to achieve a strong learner, but they are not fitted to the entire data set. The target is to minimize the errors of the previous tree. Thus, each tree fits to the residuals from the previous one, reducing the need to have correlated trees (Friedman, 2001; Hastie et al., 2009). As a result, the global accuracy and robustness of the model regularly increase. Additionally, GBM does not use or need bootstrapping.

Gradient boosting machine, as any supervised machine‐learning algorithm, works for regression and classification that produces a prediction model with reduced variance and bias in the form of an ensemble of simple prediction models (Friedman, 2001; Hastie et al., 2009). The algorithm helps in the conversion of weak learners into strong learners by combining many weak learners, as the weak learners are sequentially corrected by their predecessors, and, in the process, they are converted into strong learners. As an ensemble model, GBM comes with an easy‐to‐read and easily interpretable algorithm, making its prediction interpretations easy to handle. Gradient boosting machine is not new in the context of GS since Li et al. (2018) used it to identify a subset of single‐nucleotide polymorphism makers for the genomic prediction of breeding values, while Perez et al. (2022) used it for the prediction on complex phenotypes in outbred mice. Gradient boosting machine was also used for genomic prediction of continuous maize phenotypic traits by Westhues et al. (2021).

Recently, threshold models, a type of generalized linear mixed models with probit link function (Stroup, 2012), have been considered in GS for plant breeding. Montesinos‐Lopez et al. (2015) introduced a threshold GS model that is an extension of the genomic best linear unbiased prediction of Jarquín et al. (2014) that incorporates genotype × environment interaction; this model is denoted as TGBLUP. These authors’ results highlighted the importance of including genotype × environment interaction (capturing at least 49.42% of the total variability); when this interaction was included, the total variability explained by these models was increased in addition to the prediction accuracy, which was shown between 8 and 19% relative to models based on main effects only.

The TGBLUP model proposed by Montesinos‐López et al. (2015) is a Bayesian version of classic probit models and is very competitive in terms of prediction performance, as was shown by Montesinos‐López et al. (2019) who compared this method to DL and SVM. However, because of the fact that the TGBLUP model was built under a Bayesian framework (that uses Gibbs sampling), it requires sizeable computational resources because convergence requires considerable time when used with large data sets. Montesinos‐López et al. (2020) also proposed a maximum a posteriori threshold genomic prediction (MAPT) model for ordinal traits that is more efficient than the TGBLUP with regard to implementation time; however, it is less efficient than the TGBLUP method in terms of prediction performance.

For the previously described reasons, in this paper, we benchmarked GBM against the TGBLUP. The comparison between these two methods was done for categorical traits using four real data sets (Montesinos‐López et al., 2019, 2020). The TGBLUP method was selected for the comparison because it is one of the most efficient methods in terms of prediction performance in the context of GS for binary traits (Montesinos‐López et al., 2019). The GBM was chosen because it is very popular in many fields for producing very efficient predictions, not just for binary traits. The main objective of this study was to evaluate the prediction performance of the GBM in the context of genomic selection and compare its prediction ability against the TGBLUP method, which is popular in GS.

**Core Ideas**

- Genomic‐enabled prediction was used for categorical traits to capture data patterns in different environments.
- Two different genome‐based models were used for predicting categorical traits.
- Genome‐based prediction with genotype × environment interaction was used.

## 2 MATERIALS AND METHODS

### 2.1 Phenotypic data

We used four wheat data sets that were used by Montesinos‐López et al. (2019, 2020). These four data sets were collected by the Global Wheat Program of the International Maize and Wheat Improvement Center and belong to elite yield trials (EYTs) established in four different cropping seasons with four or five environments in each. The lines involved in each of the environments of the same year are the same, but those in different years are different lines. The EYT Data Set 1 was involved in 2013–2014 and contains 767 lines; EYT Data Set 2 was established in 2014–2015 and contains 775 lines; EYT Data Set 3 was cultivated in 2015–2016 and contains 964 lines; and EYT Data Set 4 was cultivated in 2016–2017 with 980 lines. The experimental design used was an alpha‐lattice design and the lines were sown in 39 trials each covering 28 lines and two checks in six blocks with three replications. In each data set, several traits were available for some environments and lines. In this study we evaluated two traits that were measured for each line in each environment: days to heading (DTHD, number of days from germination to 50% spike emergence) and plant height. The DTHD was discretized in four categories and plant height in two. Details of the discretization process can be found in Montesinos‐López et al. (2019). For full details of the experimental design and how the best linear unbiased estimates were computed, see Juliana et al. (2018).

In EYT 2013–2014 Data Sets 1 and 4 (EYT 2016–2017), the lines under study were evaluated in four environments, while in EYT 2014–2015 Data Set 2 and EYT 2015–2016 Data Set 3, the lines were evaluated in five environments. For EYT Data Set 1, the environments were bed planting with five irrigations (Bed5IR), flat planting and five irrigations (Flat5IR), early heat (EHT), and late heat (LHT). For EYT Data Set 2, the environments were bed planting with two irrigation levels (Bed2IR), Bed5IR, Flat5IR, EHT, and LHT. For EYT Data Set 3, the environments were Bed2IR, Bed5IR, Flat5IR, flat planting with drip irrigation (FlatDrip), and LHT. Finally, for EYT Data Set 4, the environments were Bed2IR, EHT, Flat5IR, and FlatDrip.

### 2.2 Genotypic data

Genome‐wide markers for the 3,486 (667 + 775 + 964 + 980) lines in the four data sets were obtained using genotyping‐by‐sequencing (Elshire et al., 2011; Poland et al., 2012) at Kansas State University with an Illumina HiSeq2500. After filtering, 2,038 markers were obtained from an initial set of 34,900 markers. The imputation of missing markers data was carried out using LinkImpute (Money et al., 2015) and implemented in TASSEL v5 (Bradbury et al., 2007). Lines that had >50% of missing data were removed, and 2,506 lines were used in this study (767 lines in the first data set, 775 lines in the second data set, 964 lines in the third data set, and 980 lines in the fourth data set). It is also important to point out that a high level of relatedness by pedigree or kinship is expected between lines within a year of testing and also across years of testing because of the nature of the lines under study.

## 3 DATA AVAILABILITY

Details of the phenotypic and genomic data of the seven data sets used in this study can be downloaded (https://data.cimmyt.org/dataset.xhtml?persistentId =hdl:11529/10548140). Note that Data Sets 1–4 used in this article correspond to Data Sets 1–4 of Montesinos‐López et al. (2019).

### 3.1 Statistical methods

#### 3.1.1 Bayesian threshold genomic best linear unbiased prediction

The ordinal probit model assumes that based on environments (*E_i_*), genotypes (*g_j_*), and the genotype × environment interaction (ge*_ij_*), *Y_ij_* is a random variable that takes values *c* = 1,…,*C* with the following probabilities:

(1) $$\begin{array}{ccc} P\left(Y_{ij}=c\right) & = & \Phi \left(\gamma_{c}+E_{i}+g_{j}+ge_{ij}\right)\\ & & -\Phi \left(\gamma_{c-1}+E_{i}+g_{j}+ge_{ij}\right),c=1,\text{…},C \end{array}$$

where *E_i_* are the fixed effects of environment *i* = 1, …, *I*. For this reason, the beta coefficients were estimated for environments, where *g_j_*, *j* = 1, …, *J*, are the random effects of lines distributed as $N(0,G\sigma_{g}^{2})$, and **G** is the genomic relationship matrix computed as suggested by VanRaden (2008); $\sigma_{g}^{2}$ is the genetic variance component, ge*_ij_* is the genotype × environment interaction term distributed as $N\left(0,I_{I}⊗G\sigma_{\mathrm{ge}}^{2}\right),\sigma_{\mathrm{ge}}^{2}$ is the corresponding variance component for the genotype × environment interaction term, and $-\infty =\gamma_{0}<\gamma_{1}<\cdots <\gamma_{C}=\infty$ are threshold parameters. A Bayesian formulation of this model assumes the following independent priors for the parameters: a flat prior distribution for $\gamma =\left(\gamma_{1},\cdots ,\gamma_{C-1}\right)\left[f\left(\gamma \right)\propto 1\right],$ a normal distribution for beta coefficients of fixed effects $E_{i}∼N\left(0,10^{10}\right),i=1,\cdots ,I-1$, and a scale inverse chi‐squared distribution for $\sigma_{g}^{2}\left(\sigma_{g}^{2}∼\chi_{v_{g},S_{g}}^{-2}\right)$ and $\sigma_{\mathrm{ge}}^{2}\left(\sigma_{\mathrm{ge}}^{2}∼\chi_{v_{\mathrm{ge}},S_{\mathrm{ge}}}^{-2}\right)$ (Montesinos‐López et al., 2015; Pérez‐Rodríguez & de los Campos, 2014).

This threshold model assumes that the process that gives rise to the observed categories is an underlying or latent continuous normal random variable $l_{ij}=-E_{i}-g_{j}-ge_{ij}+\varepsilon_{i}$ where ε*_i_* is a normal random variable with mean 0 and variance 1, and the values of *l_i_* are called ‘liabilities’ (Gianola, 1982; Sorensen et al., 1995). The ordinal categorical phenotypes in Equation 1 (Model 1) are generated from the underlying phenotypic values, *l_i_*, as follows: $Y_{\mathrm{ij}}=1\mathrm{if}-\infty <l_{\mathrm{ij}}<\gamma_{1},Y_{\mathrm{ij}}=2$ if $\gamma_{1}<l_{\mathrm{ij}}<\gamma_{2},\cdots ,\mathrm{and}Y_{\mathrm{ij}}=C\mathrm{if}\gamma_{C-1}<l_{\mathrm{ij}}<\infty$. The TGBLUP model can be implemented in the BGLR package of Pérez‐Rodríguez and de los Campos (2014) in the R statistical software (R Core Team, 2021).

#### 3.1.2 Gradient boosting machine

The GBM is a powerful machine‐learning algorithm that has been used in a wide range of data driven applications in fields such as ecology, computer science, biology, and genomic prediction problems. Friedman (2001) proposed a modification to the original gradient boosting algorithm. The author observed improvement in gradient boosting's accuracy by proposing fitting a base learner on a subsample of the training set drawn at random without replacement at each iteration of the algorithm. Friedman (2001) developed the boosting paradigm based on various fitting criteria and named this regression technique GBM. The author concluded that this approach is competitive and robust for interpreting regression of nonclean data.

We implemented the following GBM algorithm proposed by Friedman (2001):

**Inputs**:

input data (*y_i_*, *x_i_*), for *i* = 1, 2, …, *n*
number of iterations *M*
choice of the loss‐function φ(*y*, *f*)
choice of the base‐learner model *h*(*x*, θ)

**Algorithm**:

Step 1: initialize *f*_0_ with a constant
Step 2: for *t* = 1 to *M* repeat Steps 3–6:
Step 3: compute the negative gradient of φ(*y_i_*, *f*) with respect to *f*: *g_t_* (*x_i_*)
Step 4: fit a new base‐learner function *h*(*x*, θ*_t_*) for predicting *g_t_* (*x_i_*) from the covariables *x_i_*
Step 5: find the best gradient descent step‐size ρ*_t_*:
$$\rho_{t}=\arg\min_{\rho }\sum_{i=1}^{n}\varphi \left[y_{i},{\hat{f}}_{t-1}\left(x_{i}\right)+\rho h\left(x_{i},\theta_{t}\right)\right]$$
Step 6: update the function estimate: ${\hat{f}}_{t}\leftarrow {\hat{f}}_{t-1}+\rho_{t}h\left(x_{i},\theta_{t}\right)$
Step 7: final predictions:$\hat{f}\left(x\right)={\hat{f}}_{M}\left(x\right)$

The GBM method was implemented using the Bernoulli loss functions (based on Bernoulli distribution), where the decision trees with interaction depth equal to three were used as a base‐learner model *h*(*x*, θ*_t_*). Three thousand trees were grown, the value of shrinkage used was 0.1 and the minimum number of observations in the terminal nodes of the tree was set to 5. We used the GBM library to implement it in this study (Greenwell et al., 2020). Similarly, as in the TGBLUP method, the information used as independent variables contains information from environments plus the information of the genotypes with markers and the information of the genotype by environment interaction. However, in the TGBLUP method, the information from environments, genotypes, and genotype × environment interactions are introduced as separate blocks of information (see Equation 1). Contrarily, under the gradient boosting machine, this information is concatenated and included in only a matrix of inputs $X=[X_{E},X_{G},X_{\mathrm{GE}}]$, where **X_E_** denotes the design matrix of environments, **X_G_** denotes the design matrix of genotype postmultiplied by the square root of the genomic relationship matrix, and **X_GE_** denotes the design matrix of the genotype × environment interaction that also takes into account the square root of the genomic relationship matrix. This implied that each row (*x_i_* for *i* = 1, 2, …, *n*) of the input matrix **X** was used in the GBM method. For more specific details of GBM, see Friedman (2001).

### 3.2 Evaluation of prediction performance

We used 30 random partitions to evaluate the prediction performance in each data set. Random partitions are a type of cross‐validation where the data are divided into training and testing at random, and the user specifies the proportion of data assigned to training and testing. In our case, we used, in each partition, 20, 40, 60, and 80% for training and their corresponding complements for testing (80, 60, 40, and 20%). The model was fitted with the training set, and we evaluated the prediction performance with the testing set. Each partition (for example, with training = 80% of the data and testing = 20% of the data) was repeated 30 times and the average prediction performance of the 30 random partitions was reported as prediction performance. In half of the training–testing partitions used; the training set is smaller than the testing set. The evaluation was carried out in this way because, in real applications, we are interested in only using a small training set to predict a large testing set. While these scenarios may seem extreme, the only restriction from a statistical point of view is that we frequently have too little data to estimate the required parameters with enough precision. With the information from each testing set (observed and predicted), we computed the proportion of cases correctly classified, which is also known as ‘accuracy’, $\mathrm{PCCC}=\left(\mathrm{tp}+\mathrm{tn}\right)/n_{T}$, where tp denotes the true positives, tn denotes the true negatives, and $n_{T}=\left(\mathrm{tp}+\mathrm{tn}+\mathrm{fp}+\mathrm{fn}\right)$ is the total of individuals in the testing sets, where fp denotes the false positives, and fn denotes the false negatives (Fielding & Bell, 1997; González‐Camacho et al., 2018; ISO 5725–6, 1994; Montesinos‐López, et al., 2019; Montesinos‐López, et al., 2020). The true positives or true negatives are outcomes where the model correctly predicts them. Similarly, false positives are outcomes where the model incorrectly predicts the positives, and the false negatives are outcomes where the model incorrectly predicts the negatives. All components for computing the proportion of cases correctly classified (PCCC) are obtained from a confusion matrix obtained between observed and predicted values (Fielding & Bell, 1997; González‐Camacho, 2018) and the Kappa coefficient proposed by Cohen (1960) for the agreement for nominal scales, $\mathrm{Kappa}=\left(PCCC-P_{e}\right)/\left(1-P_{e}\right)$; *P_e_* is the probability of agreement calculated as $P_{e}=\frac{\mathrm{tp}+\mathrm{fn}}{n_{T}}\times \frac{\mathrm{tp}+\mathrm{fp}}{n_{T}}\times \frac{\mathrm{fp}+\mathrm{tn}}{n_{T}}\times \frac{\mathrm{fn}+\mathrm{tn}}{n_{T}}$. These are two popular metrics used to evaluate prediction performance in binary and categorical traits (Fielding & Bell, 1997; González‐Camacho, 2018).

Under both metrics, the closer to one, the better the predictions. In the case of the Kappa coefficient, the interpretation is as follows: values < 0.00 as indicating no agreement, 0.0–0.20 as none to slight, 0.21–0.40 as fair, 0.41– 0.60 as moderate, 0.61–0.80 as substantial, and 0.81–1.00 as almost perfect agreement. Finally, the average of the 30 partitions of both metrics was reported as the prediction performance for each environment in each data set under study. We used the R statistical software for the implementation of both prediction models (R Core Team, 2021). For the categorical response variable (DTHD), we also computed the PCCC and the Kappa coefficient; however, the Kappa coefficient was computed using the one vs. all approach (James et al., 2013). This means that it was computed for each of the four categories vs. the aggregation of the remaining categories, and the average of four scores of Kappa coefficient was reported as prediction performance.

## 4 RESULTS

The most important findings are provided in the following three sections. The first two sections show results for Data Sets 3 and 4, while the third section provides a meta‐picture of the prediction performance of the four data sets averaged across environments. We have first included the results from Data Sets 3 and 4, which represent the two largest data sets with 964 lines (Data Set 3) and 980 wheat lines (Data Set 4). Note that the results obtained from Data Sets 1 and 2 followed similar patterns as those from Data Sets 3 and 4 but are presented separately in Supplemental Figures S1–S12 for the purpose of simplifying the description of the results.

### 4.1 Data Set 3

#### 4.1.1 Binary trait (height)

**FIGURE 1:**
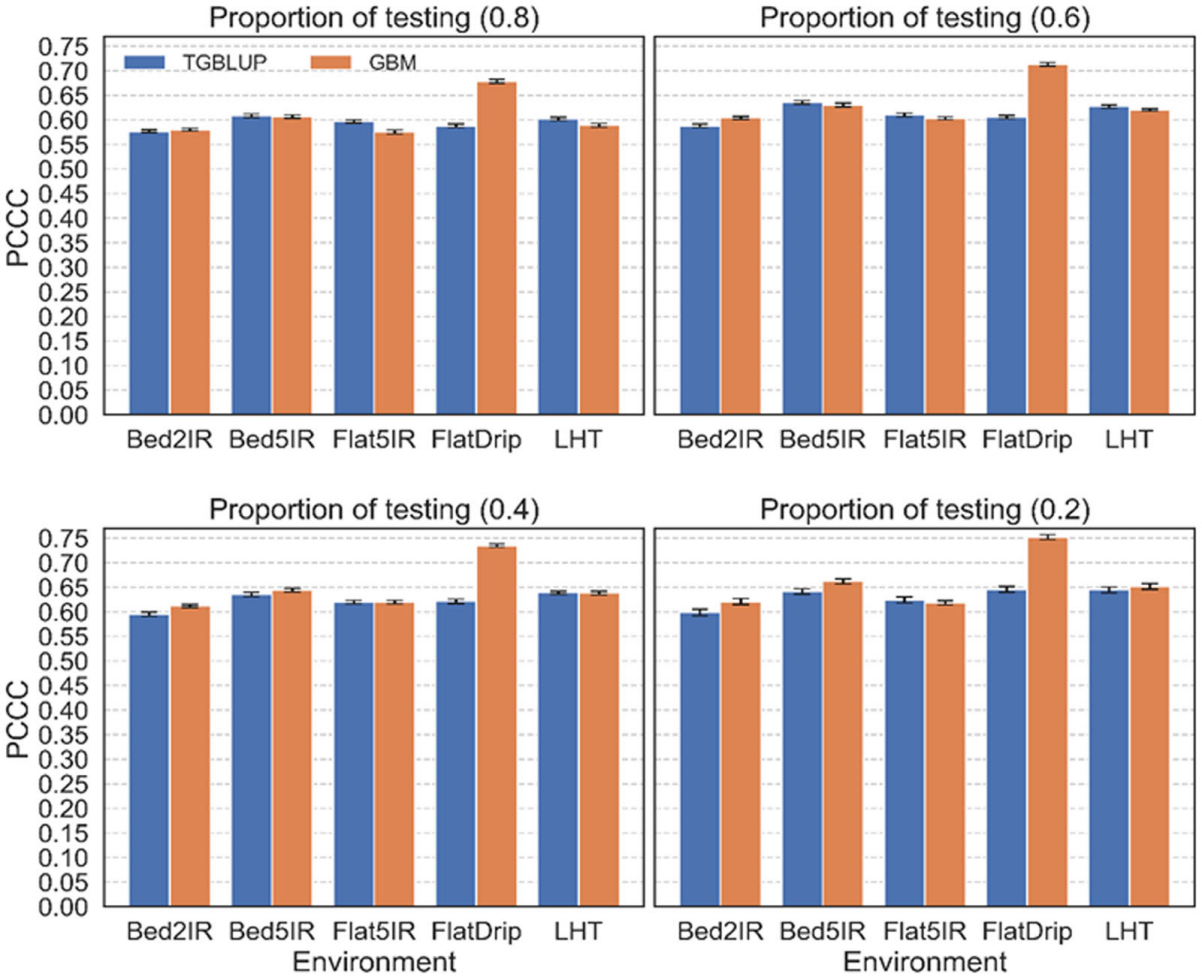
Data Set 3. Prediction performance of the height (binary) trait under the threshold genomic best linear unbiased predictor model (TGBLUP) and gradient boosting machine (GBM) for four proportions of testing sets (0.2, 0.4, 0.6, and 0.8) for each environment in terms of the proportion of cases correctly classified (PCCC). Five environments: Bed2IR (bed planting system under two irrigations), Bed5IR (bed planting system under five irrigations), Flat5IR (flat planting system under five irrigations), FlatDrip (flat planting system under drought), and LHT (late heat planting). The whisker plots indicate the standard errors

Figure 1 shows that the GBM outperformed the TGBLUP method in terms of PCCC only in two out of the five environments for two proportions of the testing sets (0.8 and 0.6). Specifically, when the proportion of the testing set was 0.8, the GBM outperformed the TGBLUP method in the Bed2IR and FlatDrip environments by 0.57 and 15.29%, respectively. The TGBLUP method outperformed the GBM in the three remaining environments (Bed5IR, Flat5IR, and LHT by 0.27, 3.60, and 2.18%, respectively). Likewise, when the proportion of the testing set was 0.6, the GBM outperformed TGBLUP in the Bed2IR and FlatDrip environments by 2.73 and 17.76%, respectively. The TGBLUP method outperformed the GBM in the three remaining environments (Bed5IR, Flat5IR, and LHT by 0.75, 1.10, and 1.03%, respectively).

The GBM performance improved for the proportions of testing sets 0.4 and 0.2, in which the GBM outperformed the TGBLUP method in four out of the five environments. When the proportion of the testing set was 0.4, the GBM outperformed the TGBLUP method in the Bed2IR, Bed5IR, Flat5IR, and FlatDrip environments by 2.83, 1.43, 0.01, and 18.28%, respectively, while the TGBLUP method only outperformed the GBM in the LHT environment by 0.05%. When the proportion of the testing set was 0.2, the GBM outperformed the TGBLUP method in the Bed2IR, Bed5IR, FlatDrip, and LHT environments by 3.68, 3.24, 16.51, and 1.131%; the TGBLUP method only outperformed the GBM in the Flat5IR environment by 0.30%. Furthermore, the GBM outperformed the TGBLUP method in all of the proportions of testing sets across all environments (by 2.24, 3.61, 4.86, and 5.05% when the proportion of the testing set was 0.8, 0.6, 0.4, and 0.2, respectively).

**FIGURE 2:**
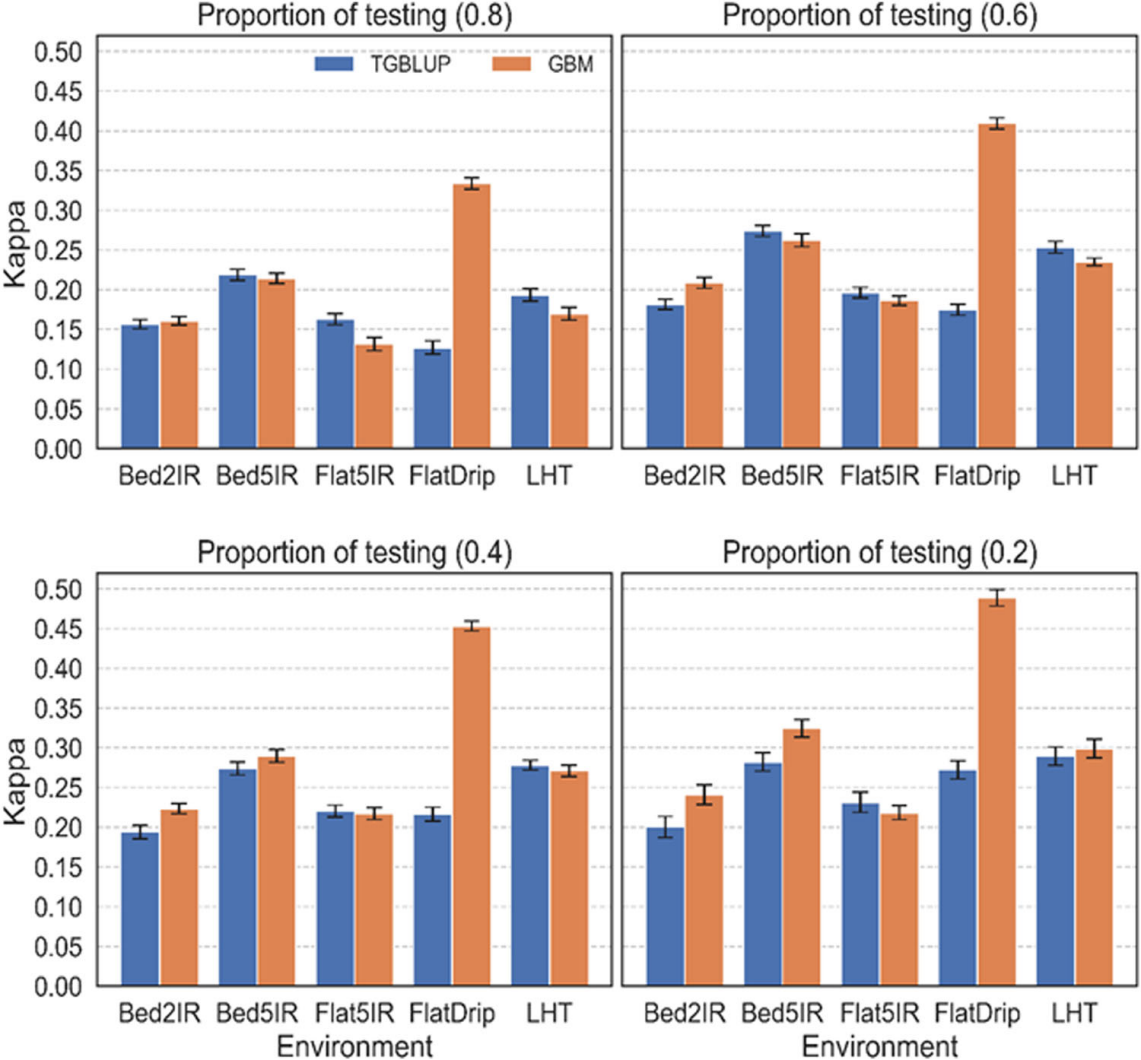
Data Set 3. Prediction performance of the height (binary) trait under the threshold genomic best linear unbiased predictor model (TGBLUP) and gradient boosting machine (GBM) for four proportions of testing sets (0.2, 0.4, 0.6, and 0.8) for each environment in terms of the Kappa coefficient (Kappa). Five environments: Bed2IR (bed planting system under two irrigations), Bed5IR (bed planting system under five irrigations), Flat5IR (flat planting system under five irrigations), FlatDrip (flat planting system under drought), and LHT (late heat planting). The whisker plots indicate the standard errors

In Figure 2, in terms of the Kappa coefficient, we observed behavior similar to that described above. When the proportion of the testing set was 0.8, the GBM outperformed the TGBLUP method in the Bed2IR and FlatDrip environments by 2.74 and 162.44%, respectively. The TGBLUP method outperformed the GBM in the three remaining environments (Bed5IR, Flat5IR, and LHT by 2.11, 19.27, and 12.29%, respectively). Likewise, when the proportion of the testing set was 0.6, the GBM outperformed TGBLUP in the Bed2IR and FlatDrip environments by 14.89 and 134.08%, respectively. The TGBLUP method outperformed the GBM method in the three remaining environments (Bed5IR, Flat5IR, and LHT by 4.3, 5, and 7.21%, respectively).

The GBM performance improved for the proportions of testing sets 0.4 and 0.2, in which the GBM outperformed the TGBLUP method in three and four out of the five environments, correspondingly. When the proportion of the testing set was 0.4, the GBM outperformed the TGBLUP method in the Bed2IR, Bed5IR, and FlatDrip environments by 15.03, 5.69, and 109.28%, respectively. The TGBLUP method outperformed the GBM in the Flat5IR and LHT environments by 1.34 and 2.57, respectively. When the proportion of the testing set was 0.2, the GBM outperformed the TGBLUP method in the Bed2IR, Bed5IR, FlatDrip, and LHT environments by 20.11, 14.89, 79.55, and 3.29%, respectively; the TGBLUP method only outperformed the GBM in the Flat5IR environment by 3.98%.

#### 4.1.2 Categorical trait (DTHD)

In the categorical trait DTHD, Table 1 shows that the GBM outperformed the TGBLUP method in terms of PCCC in all five environments (Bed2IR, Bed5IR, Flat5IR, FlatDrip, and LHT) for all the proportions of testing sets. When the proportion of the testing set was 0.8, we observed that the GBM outperformed the TGBLUP method by 6.57, 1.92, 3.66, 6.45, and 22.53% in environments Bed2IR, Bed5IR, Flat5IR, FlatDrip, and LHT, respectively. When the proportion of the testing set was 0.6, the GBM outperformed the TGBLUP method by 7.19, 3.83, 12.6, 7.82, and 37.97% in environments Bed2IR, Bed5IR, Flat5IR, FlatDrip, and LHT, respectively. When the proportion of the testing set was 0.4, the GBM outperformed the TGBLUP method by 8.38, 12.12, 18.22, 8.77, and 56.05% in environments Bed2IR, Bed5IR, Flat5IR, FlatDrip, and LHT, respectively. When the proportion of the testing set was 0.2, the GBM outperformed the TGBLUP method by 11.74, 16.73, 21.78, 8.34, and 57.49 in environments Bed2IR, Bed5IR, Flat5IR, FlatDrip, and LHT, respectively.

**TABLE 1:** Prediction performance for each environment at different proportions of the testing sets (PropTesting that is predicted 0.8–0.2) in terms of the proportion of cases correctly classified (PCCC) and the Kappa coefficient (Kappa) for Data Sets 3 and 4 for the categorical trait days to heading

			PCCC		Kappa	
Data Set	PropTesting	Environment	TGBLUP ± SE	GBM ± SE	TGBLUP ± SE	GBM ± SE
3	0.8	Bed2IR	0.268 ± 0.003	0.285 ± 0.002	0.117 ± 0.007	0.100 ± 0.008
		Bed5IR	0.296 ± 0.003	0.302 ± 0.003	0.129 ± 0.006	0.145 ± 0.007
		Flat5IR	0.281 ± 0.003	0.291 ± 0.003	0.064 ± 0.007	0.072 ± 0.007
		FlatDrip	0.267 ± 0.003	0.285 ± 0.003	0.097 ± 0.008	0.104 ± 0.008
		LHT	0.378 ± 0.006	0.463 ± 0.003	0.142 ± 0.009	0.164 ± 0.007
		Average	0.298 ± 0.004	0.325 ± 0.003	0.110 ± 0.008	0.117 ± 0.007
	0.6	Bed2IR	0.289 ± 0.002	0.310 ± 0.003	0.153 ± 0.007	0.140 ± 0.007
		Bed5IR	0.319 ± 0.003	0.331 ± 0.003	0.178 ± 0.007	0.201 ± 0.008
		Flat5IR	0.277 ± 0.004	0.312 ± 0.003	0.092 ± 0.007	0.097 ± 0.009
		FlatDrip	0.290 ± 0.004	0.313 ± 0.003	0.152 ± 0.006	0.143 ± 0.007
		LHT	0.353 ± 0.004	0.487 ± 0.003	0.173 ± 0.007	0.200 ± 0.008
		Average	0.306 ± 0.003	0.351 ± 0.003	0.150 ± 0.007	0.156 ± 0.008
	0.4	Bed2IR	0.293 ± 0.003	0.318 ± 0.003	0.162 ± 0.008	0.143 ± 0.009
		Bed5IR	0.315 ± 0.004	0.353 ± 0.004	0.175 ± 0.006	0.222 ± 0.010
		Flat5IR	0.274 ± 0.004	0.324 ± 0.004	0.102 ± 0.010	0.112 ± 0.009
		FlatDrip	0.301 ± 0.004	0.327 ± 0.003	0.164 ± 0.008	0.173 ± 0.008
		LHT	0.328 ± 0.006	0.512 ± 0.004	0.179 ± 0.009	0.236 ± 0.010
		Average	0.302 ± 0.004	0.367 ± 0.004	0.156 ± 0.009	0.177 ± 0.009
	0.2	Bed2IR	0.293 ± 0.006	0.327 ± 0.005	0.162 ± 0.013	0.125 ± 0.014
		Bed5IR	0.310 ± 0.006	0.362 ± 0.007	0.170 ± 0.013	0.229 ± 0.015
		Flat5IR	0.273 ± 0.004	0.332 ± 0.005	0.112 ± 0.013	0.106 ± 0.014
		FlatDrip	0.304 ± 0.005	0.329 ± 0.006	0.170 ± 0.012	0.181 ± 0.013
		LHT	0.334 ± 0.005	0.526 ± 0.006	0.181 ± 0.011	0.251 ± 0.015
		Average	0.303 ± 0.005	0.375 ± 0.006	0.159 ± 0.012	0.178 ± 0.014
4	0.8	Bed5IR	0.296 ± 0.004	0.290 ± 0.002	0.102 ± 0.007	0.096 ± 0.008
		EHT	0.299 ± 0.003	0.295 ± 0.003	0.108 ± 0.007	0.089 ± 0.007
		Flat5IR	0.278 ± 0.003	0.292 ± 0.004	0.083 ± 0.007	0.091 ± 0.007
		FlatDrip	0.300 ± 0.003	0.306 ± 0.003	0.093 ± 0.008	0.101 ± 0.008
		Average	0.293 ± 0.003	0.296 ± 0.003	0.097 ± 0.007	0.094 ± 0.008
	0.6	Bed5IR	0.310 ± 0.003	0.312 ± 0.004	0.145 ± 0.007	0.122 ± 0.008
		EHT	0.319 ± 0.002	0.318 ± 0.003	0.156 ± 0.008	0.123 ± 0.007
		Flat5IR	0.302 ± 0.003	0.313 ± 0.003	0.137 ± 0.007	0.127 ± 0.008
		FlatDrip	0.322 ± 0.004	0.328 ± 0.003	0.139 ± 0.008	0.135 ± 0.007
		Average	0.313 ± 0.003	0.318 ± 0.003	0.144 ± 0.007	0.127 ± 0.008
	0.4	Bed5IR	0.317 ± 0.005	0.326 ± 0.003	0.179 ± 0.011	0.133 ± 0.009
		EHT	0.326 ± 0.003	0.331 ± 0.004	0.178 ± 0.009	0.135 ± 0.008
		Flat5IR	0.299 ± 0.004	0.332 ± 0.004	0.150 ± 0.011	0.149 ± 0.009
		FlatDrip	0.331 ± 0.004	0.343 ± 0.004	0.163 ± 0.012	0.155 ± 0.010
		Average	0.318 ± 0.004	0.333 ± 0.004	0.168 ± 0.011	0.143 ± 0.009
	0.2	Bed5IR	0.313 ± 0.006	0.336 ± 0.006	0.178 ± 0.013	0.140 ± 0.013
		EHT	0.339 ± 0.005	0.332 ± 0.006	0.200 ± 0.016	0.131 ± 0.011
		Flat5IR	0.299 ± 0.005	0.359 ± 0.005	0.158 ± 0.014	0.200 ± 0.014
		FlatDrip	0.326 ± 0.006	0.347 ± 0.005	0.162 ± 0.012	0.158 ± 0.012
		Average	0.319 ± 0.006	0.343 ± 0.005	0.175 ± 0.014	0.157 ± 0.013

*Note*. TGBLUP, threshold genomic best linear unbiased predictor model; GBM, gradient boosting machine; Bed2IR, bed planting system under two irrigations; Bed5IR, bed planting system under five irrigations; Flat5IR, flat planting system under five irrigations; FlatDrip, flat planting system under drought; LHT, late heat planting. . The PCCC and Kappa were computed in the testing set as the average of the 30 random partitions; SE denotes the standard error. Average denotes the average performance across five (Data Set 3) and four environments (Data Set 4)

In terms of the Kappa coefficient, Table 1 indicates that, in the categorical trait DTHD, the GBM outperformed the TGBLUP method in three and four out of the five environments for the proportions of testing sets 0.2, 0.6, 0.4, and 0.8, respectively. When the proportion of the testing set was 0.6, we observed that the GBM outperformed the TGBLUP method by 12.47, 4.77, and 15.39% in environments Bed5IR, Flat5IR, and LHT, respectively. The TGBLUP method outperformed the GBM in the two remaining environments (Bed2IR and FlatDrip by 8.25 and 5.83%, respectively). When the proportion of the testing set was 0.2, the GBM outperformed the TGBLUP method by 35.05, 6.5, and 38.42% in environments Bed5IR, FlatDrip, and LHT, respectively. The TGBLUP method outperformed the GBM in the two remaining environments (Bed2IR and Flat5IR by 22.48 and 5.8%, respectively). When the proportion of the testing set was 0.4, we observed that the GBM outperformed the TGBLUP method by 26.79, 9.96, 5.53, and 31.85% in environments Bed5IR, Flat5IR, FlatDrip, and LHT, respectively. The TGBLUP method outperformed the GBM only in the remaining environment (Bed2IR by 11.51%). When the proportion of the testing set was 0.8, we observed that the GBM outperformed the TGBLUP method by 12.75, 12.51, 6.66, and 15.66% in environments Bed5IR, Flat5IR, FlatDrip, and LHT, respectively. The TGBLUP method outperformed the GBM only in the remaining environment (Bed2IR by 14.15%).

### 4.2 Data Set 4

#### 4.2.1 Binary trait (Height)

**FIGURE 3:**
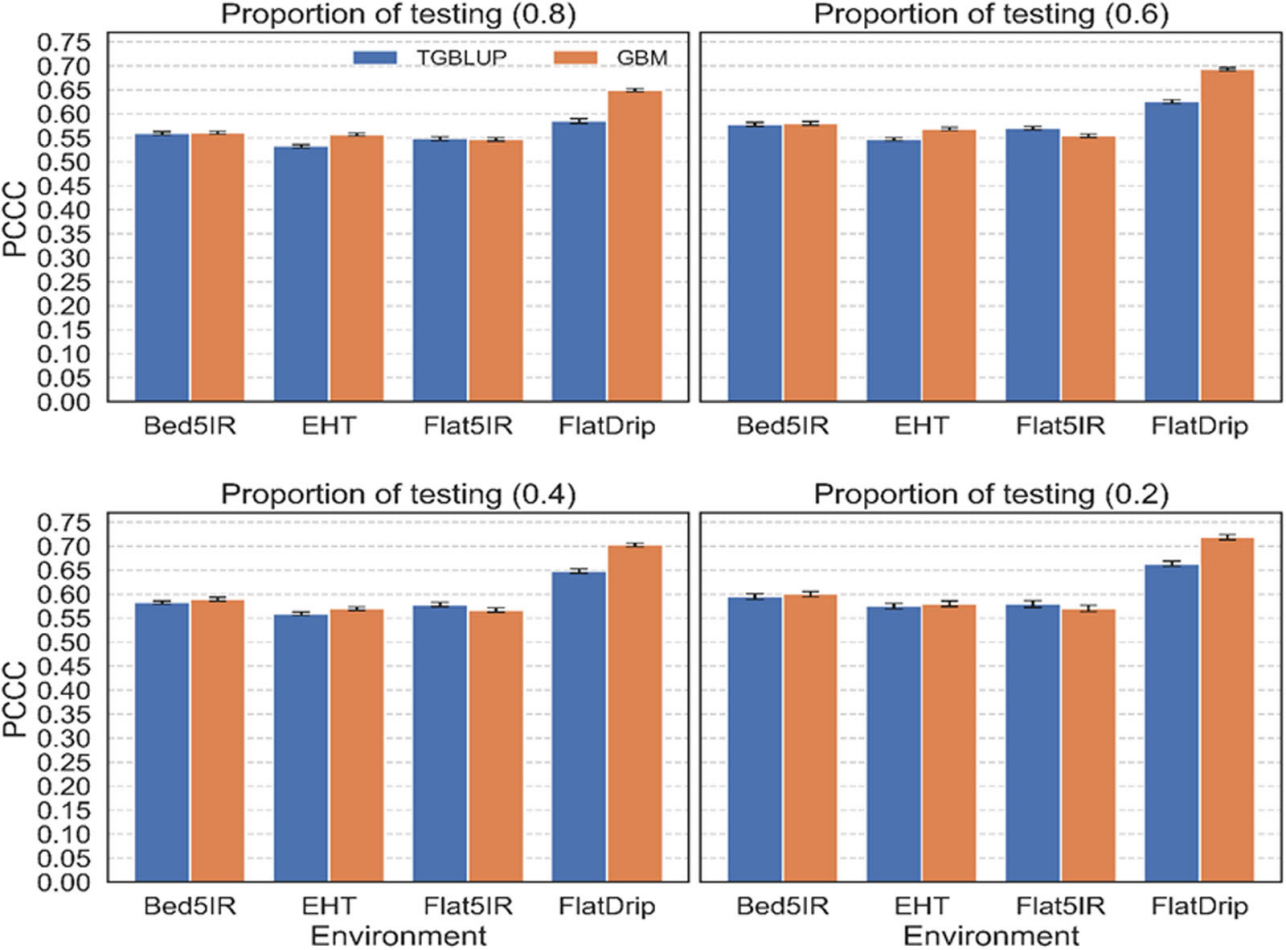
Data Set 4. Prediction performance of the height (binary) trait under the threshold genomic best linear unbiased predictor model (TGBLUP) and gradient boosting machine (GBM) under four proportions of the testing sets (0.2, 0.4, 0.6, and 0.8) for each environment in terms of the proportion of cases correctly classified (PCCC). Four environments: Bed5IR (bed planting system under five irrigations), EHT (early heat planting); Flat5IR (flat planting system under five irrigations), and FlatDrip (flat planting system under drought). The whisker plots indicate the standard errors

Figure 3 shows that the GBM outperformed the TGBLUP method in three of the four environments (EHT, Bed5IR, and FlatDrip) for all the proportions of testing sets in terms of PCCC. In the Flat5IR environment, the TGBLUP method outperformed the GBM only by 0.30, 2.84, 1.98, and 1.61% when the testing set was 0.8, 0.6, 0.4, and 0.2, respectively. On average, the GBM outperformed the TGBLUP method in all of the proportions of testing sets for all environments (by 4, 3.23, 2.54, and 2.36% when the proportion of testing set was 0.8, 0.6, 0.4, and 0.2, respectively).

**FIGURE 4:**
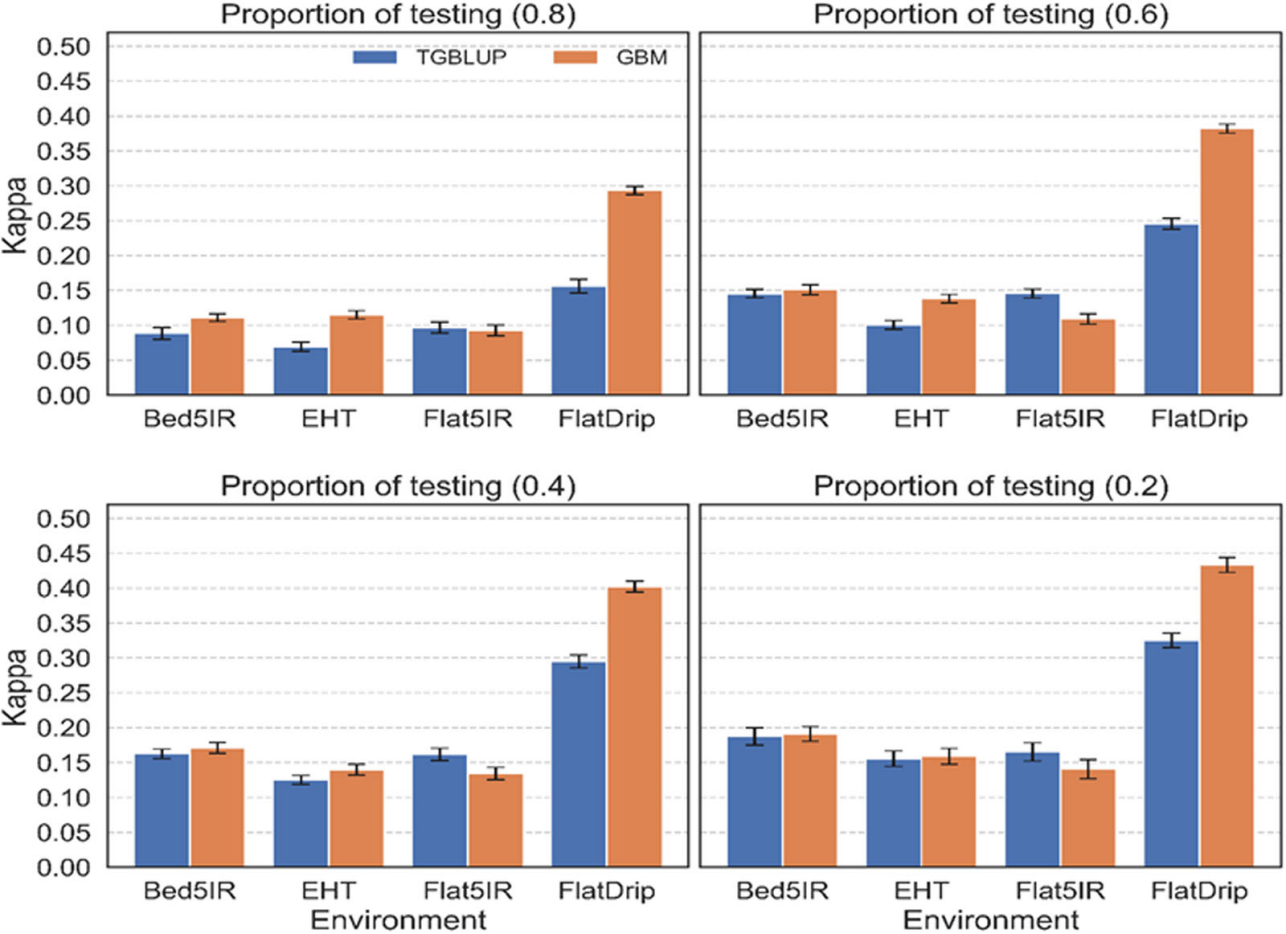
Data Set 4. Prediction performance of the height (binary) trait under the threshold genomic best linear unbiased predictor model (TGBLUP) and gradient boosting machine (GBM) under Data Set 4 for four proportions of the testing sets (0.2, 0.4, 0.6, and 0.8) for each environment in terms of the Kappa coefficient (Kappa). Four environments: Bed5IR (bed planting system under five irrigations), EHT (early heat planting); Flat5IR (flat planting system under five irrigations), and FlatDrip (flat planting system under drought). The whisker plots indicate the standard errors

In terms of the Kappa coefficient, Figure 4 indicates that the GBM outperformed the TGBLUP method in three of the four environments (EHT, Bed5IR, and FlatDrip) for all the proportions of testing sets. In the Flat5IR environment, the TGBLUP method outperformed the GBM by 49.08, 22.38, 13.91, and 10.89% when the testing sets were 0.8, 0.6, 0.4, and 0.2, respectively. Furthermore, on average, the GBM outperformed the TGBLUP method in all of the proportions of testing sets for all environments (by 49.08, 22.38, 13.91, and 10.89% when the proportion of testing set was 0.8, 0.6, 0.4, and 0.2, respectively). Because of the lack of consistency in obtaining these results, evaluation with other data sets is required to support our findings.

#### 4.2.2 Categorical trait (DTHD)

For the categorical trait DTHD, Table 1 indicates that the GBM outperformed the TGBLUP method in terms of PCCC in at least three of the four environments for three proportions of testing sets (0.6, 0.4, and 0.2). When the proportion of the testing set was 0.6, the GBM outperformed the TGBLUP method in the Bed5IR, Flat5IR, and FlatDrip environments by 0.6, 3.5, and 1.98%, respectively. Similarly, for testing set 0.2, the GBM outperformed the TGBLUP method in Bed5IR, Flat5IR, and FlatDrip environments by 7.33, 19.93, and 6.364%, respectively. The TGBLUP method only outperformed the GBM in the EHT environment by 0.14 and 2.05% for testing sets 0.6 and 0.2, respectively. Further, when the testing set was 0.4, the GBM outperformed the TGBLUP method in all four environments (Bed5IR, Flat5IR, FlatDrip, and EHT) by 2.89, 1.56, 10.97, and 3.75%, respectively. When the testing set was 0.8, the GBM outperformed the TGBLUP method in two (Flat5IR and FlatDrip) out of the four environments by 4.91 and 1.97%, respectively. The TGBLUP method outperformed the GBM in the remaining environments (Bed5IR and EHT by 1.95 and 1.37%, respectively).

In terms of the Kappa coefficient, Table 1 shows that, in the categorical trait DTHD, the GBM outperformed the TGBLUP method in two and one of the four environments for the proportions of testing sets 0.8 and 0.2, respectively. When the proportion of the testing set was 0.8, the GBM outperformed the TGBLUP method by 8.57 and 8.23% in environments Flat5IR and FlatDrip, respectively. The TGBLUP method outperformed the GBM in the two remaining environments (Bed5IR and EHT by 6.01 and 17.73%, respectively). When the proportion of the testing set was 0.2, we observed that the GBM outperformed the TGBLUP method by 26.16% in environment Flat5IR. The TGBLUP method outperformed the GBM in the remaining environments (Bed5IR, EHT, and FlatDrip by 21.08, 34.48, and 2.75%, respectively). The TGBLUP method outperformed the GBM in all the environments by 15.60, 21.03, 7.21, and 2.72% in environments Bed5IR, EHT, Flat5IR, and FlatDrip, respectively, when the proportion of the testing set was 0.6 and by 25.75, 24.32, 0.83, and 4.5% in environments Bed5IR, EHT, Flat5IR, and FlatDrip when the testing set was 0.4. A similar performance was obtained in Data Sets 1 and 2 (Supplemental Figures S1–S8).

### 4.3 Meta‐picture across environments for each data set

**FIGURE 5:**
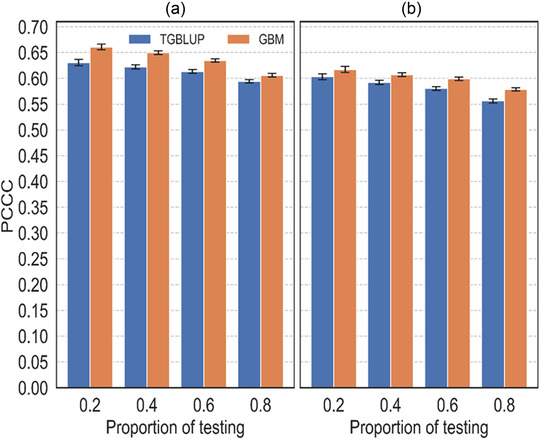
Data Sets 3 and 4. Prediction performance of the height (binary) trait under the threshold genomic best linear unbiased predictor model (TGBLUP) and gradient boosting machine (GBM) under four proportions of the testing sets (0.2, 0.4, 0.6, and 0.8) across environments in terms of the proportion of cases correctly classified (PCCC) for (a) Data Set 3 (across five environments) and (b) Data Set 4 (across four environments). The whisker plots indicate the standard errors

**FIGURE 6:**
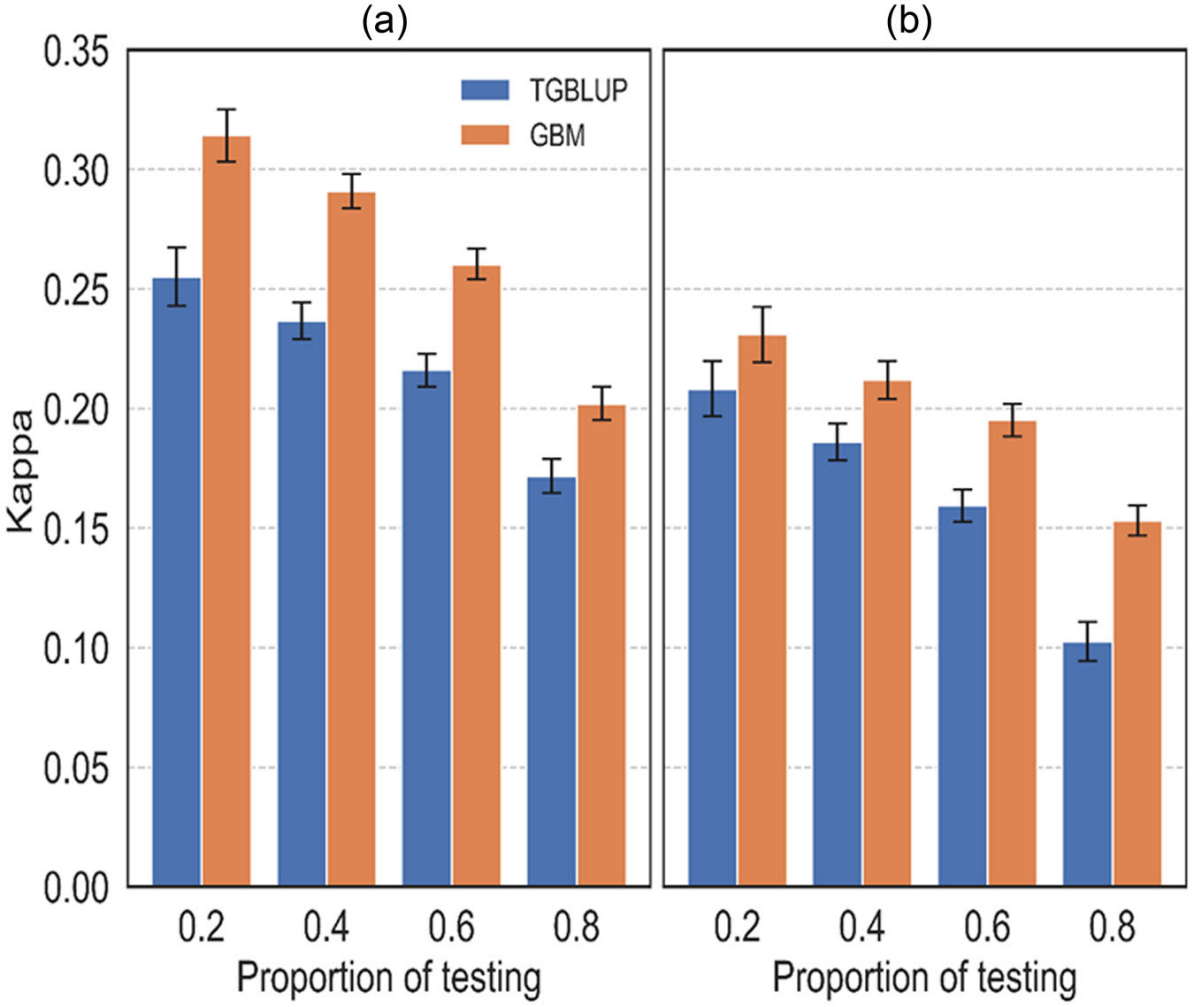
Data Sets 3 and 4. Prediction performance of the height (binary) trait under the threshold genomic best linear unbiased predictor model (TGBLUP) and gradient boosting machine (GBM) under four proportions of the testing sets (0.2, 0.4, 0.6, and 0.8) across environments in terms of the Kappa coefficient (Kappa) for (a) Data Set 3 (across five environments) and (b) Data Set 4. The whisker plots indicate the standard errors

For Data Set 3, Figure 5a and Figure 6a indicate that across environments, the GBM outperformed the TGLUP method for both metrics in all proportions of the testing sets. The GBM outperformed the TGBLUP by 2.24%, 3.61%, 4.87%, and 5.06% regarding PCCC when the proportions of testing sets were 0.8, 0.6, 0.4, and 0.2, respectively. At the same time, the GBM outperformed the TGBLUP by 20.88%, 21.66%, 24.49%, and 23.72%, respectively, under the Kappa coefficient.

For Data Set 3, for the categorical trait DTHD, Table 1 shows that across environments, the GBM outperformed the TGBLUP method in all proportions of the testing sets in terms of PCCC. The GBM outperformed the TGBLUP by 9.13%, 14.73%, 21.38%, and 23.91% for PCCC when the proportions of testing sets were 0.8, 0.6, 0.4, and 0.2, respectively. Regarding the Kappa coefficient, in Table 1, we can see that the GBM also outperformed the TGBLUP in all proportions of the testing sets. The GBM outperformed the TGBLUP by 6.67%, 4.26%, 13.37%, and 12.25% when the proportions of testing sets were 0.8, 0.6, 0.4, and 0.2, respectively.

For Data Set 4, we can observe in Figure 5b and Figure 6b that across environments, the gain in terms of prediction accuracy of the GBM with regard to the TGBLUP was considerably better for both metrics in all proportions of the testing sets. The GBM outperformed the TGBLUP by 4.001, 3.233, 2.541, and 2.361% for PCCC and by 49.087, 22.387, 13.91, and 10.895% under the Kappa coefficient when the proportions of the testing sets were 0.8, 0.6, 0.4, and 0.2, respectively.

For Data Set 4, in terms of PCCC, we can see in Table 1 that, across environments, the GBM outperformed the TGLUP method in all proportions of the testing sets. The GBM outperformed the TGBLUP by 0.85, 1.44, 4.63, and 7.58% for PCCC when the proportions of testing sets were 0.8, 0.6, 0.4, and 0.2, respectively. In addition, in Table 1, we can see that the TGBLUP outperformed the GBM method in all proportions of the testing sets. The TGBLUP outperformed the GBM by 2.39, 13.81, 17.13, and 11.07% regarding the Kappa coefficient when the proportions of the testing sets were 0.8, 0.6, 0.4, and 0.2, respectively. Quite similar behavior was observed across environments for Data Set 1 (Supplemental Figures S9a and S10a) and Data Set 2 (see Supplemental Figures S9b and S10b).

## 5 DISCUSSION

The GS methodology can be used with some candidate individuals without the need for phenotypic information. In this vein, a reference population containing phenotypic and genomic information is used to train a statistical machine‐learning model that makes predictions for candidate lines for which only genotypic information is available. However, the selection process of the best candidates using GS is very challenging and can present the following problems: (a) the data sets for training the statistical machine‐learning models are frequently small (few genotypes), (b) the phenotypic records are very noisy, (c) the traits of interest to be predicted (such as grain yield) are very complex, (d) the training and testing sets are frequently unrelated (belong to the same distribution), (e) the amount and quality of independent variables (markers, environmental information) are insufficient, (f) the records for each line in the training set have few repetitions, or (g) the models for particular data sets are often not optimal.

For the above reasons, plant breeders are interested in exploring different options to improve the prediction performance of the models used in this area (Montesinos‐López et al., 2019). For example, in the predictor, environmental covariates can be included to help improve the predictions of new lines (Jarquín et al., 2014). They are evaluating different strategies for building the training testing set to increase the prediction performance (Isidro et al., 2015). To help the predictions, they measure the spectral data of the plants and other omics data to be taken into account in the predictor. They mix genomic and pedigree information to increase the prediction accuracy. They are also adopting novel statistical machine‐learning methods to be evaluated in GS, among others. For this reason, with the goal of contributing to the efficiency of GS methodology, we performed a benchmarking between the Bayesian TGBLUP and the GBM. These two methods were compared for binary and categorical response variables.

Under the empirical comparisons using four real data sets, we found that the GBM method outperformed the TGBLUP models in terms of PCCC and the Kappa coefficient; however, the superiority of the GBM over the TGBLUP was clearer in Data Sets 2, 3, and 4 and under the Kappa coefficient. For example, in the binary response variable, the superiority of the GBM over the TGBLUP was between 3.35 and 6.68% (in Data Set 2), between 20.88 and 24.4% (in Data Set 3), and between 13.91 and 49.087% in Data Set 4. Also, in terms of implementation time, we found for the binary trait that the TGBLUB was 1,780 (Data Set 1), 1,487 (Data Set 2), 1,643 (Data Set 3), and 1,883 (Data Set 4) times slower than the GBM method. These results are very promising since, empirically, they show that the GBM is a powerful statistical machine‐learning algorithm that should be adopted for genomic prediction because it can help to increase the efficiency of the GS methodology for binary and categorical traits. An advantage of GBM over TGBLUP is that the GBM method is an ensemble (a combination of many trees) nonparametric machine‐learning method and for this reason, is more powerful for capturing complex, nonlinear patterns more efficiently; it helps reduce the variability of the resulting predictions (Friedman, 2001). However, with only four real data sets, there is not enough empirical evidence to have a complete picture of the prediction power of GBM for binary and categorical traits. For this reason, we encourage other scientists to carry out more applications of GBM on more than binary and categorical traits to accumulate more empirical evidence of the prediction ability of this method. Because GBM is simple and cheap to implement—since a complex tuning process is not required—we can experiment with many different model designs.

In the binary trait (height) and data sets (2, 3 and 4) where the GBM outperformed the TGBLUP method by a large margin, we can speculate that this difference is due to the nature of the trait and of these data sets, that is, the data sets under this trait have more complex nonlinear patterns that are more difficult to capture for linear models such as the TGBLUP method. Because GBM is a nonlinear method built with an ensemble of decision trees, it more easily captures complex nonlinear patterns. However, when these nonlinear patterns are not present in a data set, conventional linear models can be equally as sufficient for obtaining decent prediction performance.

Our findings are promising for GS because they coincide with the idea that a small change in algorithm can significantly increase the prediction performance of specific traits. This is key for GS since improved predictions lead to more accuracy with this predictive methodology called GS. Nevertheless, to be able to select the best algorithm for a specific data set, we need to evaluate more than one machine‐learning algorithm, which is supported by the statement of the ‘no‐free‐lunch’ theorem that says that there is no universal machine‐learning algorithm. This is the same as saying that any two optimization algorithms are equivalent when their performance is averaged across all possible problems (Wolpert & Macready, 2005). Although the GBM method is not new in the machine‐learning community, it is promising for GS since it can help increase the model options available for the GS methodology and GS applications because of the ease in understanding the fundamentals and implementation of this method. Furthermore, it has the power to capture nonlinear patterns that are not easy to capture with most parametric genomic prediction models. Nonetheless, we need to be careful in GBM implementation; even though it is possible to incorporate all the available information (genomics, environmental, pedigree, etc.) in the predictor, this does not always guarantee the best out‐of‐sample predictions. It is also important to select the right loss function depending on the type of outcomes. The most popular loss function for continuous outcomes is the mean square error; for binary data, it is the Bernoulli; for categorical response, it is the binomial loss function; while for other families, users need to select or create custom loss function (Natekin & Knoll, 2013). Likewise, because GBM can be implemented with any base learners, the selection of these is important. Many base learners exist for GBM, and the most common can be classified as linear models, smooth models, and decision trees. In our application, while we used decision trees as base learners, we also used linear models, such as penalized ridge regression, ordinary linear regression of random effects models, and even a mixture of base‐learners, which can be very helpful in some applications. However, not all available libraries that implement GBM offer so much flexibility (Natekin & Knoll, 2013). For this reason, users usually only implement the available options that are in the selected library.

It is important to point out that in our benchmarking between the TGBLUP and GBM, we included the information of environment, genotypes, and genotype × environment interaction in the predictor of both models. Nevertheless, it is possible to include additional information or even omit the genotype × environment interaction. In our case, we included the genotype × environment interaction since in plant breeding, the term is frequently very important in explaining a considerable proportion of total variability. However, since the GBM is a nonlinear model, it may not require to explicitly input this information (genotype × environment interaction) without having significant loss of prediction power. For this reason, these issues can be of interest for further research.

The GBM has been applied in many areas and used to tackle various statistical machine‐learning challenges (Bissacco et al., 2007; Hutchinson et al., 2011; Pittman & Brown, 2011; Johnson & Zhang, 2014). Additionally, as pointed out in the introduction, this method had been implemented in GS for the prediction of continuous traits in plant breeding for maize phenotypic traits (Li et al., 2018) and in animal science for body weight phenotypes of Brahman cattle (Westhues et al., 2021), as well as for the prediction of complex phenotypes in outbred mice (Perez et al., 2022). Additionally, GBM is very attractive for genomic prediction since it is more efficient in the context of ‘large *p* and small *n*’ and works not only for binary and categorical outcomes but is also a powerful supervised learning algorithm able to learn complex nonlinear functions to solve regression problems. Finally, we need to understand GBM as a family of powerful machine‐learning techniques that have shown substantial success in a wide range of practical applications, in part, because they are highly customizable to the particular needs of the applications (Naketin & Knoll, 2013). Furthermore, we encourage the exploration of variants of the GBM, like the lightGBM and XGBoost, that have had great success both in enterprise applications and data science competitions (Naketin & Knoll, 2013). A disadvantage of GBM, as most machine‐learning methods, is that they cannot estimate genetic parameters like TGBLUP and most parametric models.

## 6 CONCLUSIONS

We found that, in terms of PCCC and the Kappa coefficient, the GBM outperformed the TGBLUP method in the four data sets. The superiority of the GBM over the TGBLUP method was larger in terms of the Kappa coefficient than the PCCC. The power of GBM is attributed to the fact that it is a nonlinear prediction model in the form of an ensemble of weak prediction models, typically decision trees. We encourage researchers making other applications in the context of GS to be able to accumulate more empirical evidence of GBM's power and fully consider including the GBM in the toolkit of breeding scientists for predicting binary and categorical traits in GS. Nevertheless, in this application, based on binary and categorical traits, we observed a considerable gain in terms of prediction performance of the GBM over the TGBLUP method.

## AUTHOR CONTRIBUTIONS

Osval Antonio Montesinos‐López: Conceptualization; Formal analysis; Investigation; Methodology; Validation; Writing – original draft; Writing – review & editing. Henry Nicole Gonzalez: Formal analysis; Investigation. Abelardo Montesinos‐López: Conceptualization; Formal analysis; Investigation; Methodology; Validation; Writing – original draft. María Daza‐Torres: Formal analysis; Investigation; Methodology; Writing – review & editing. Morten Lillemo: Methodology; Writing – review & editing. Jose Montesinos‐López: Conceptualization; Formal analysis; Investigation; Methodology; Writing – review & editing. Jose Crossa: Conceptualization; Investigation; Methodology; Writing – review & editing.

## CONFLICT OF INTEREST

The authors declare no conflict of interest.

## Supporting information

Supplemental Figure S1. Prediction performance of the height (binary) trait under the TGBLUP and GBM under Data Set 1 for four proportions of the testing sets (0.2, 0.4, 0.6 and 0.8) for each environment in terms of the proportion of cases correctly classified (PCCC). The whisker plots indicate the standard errors.

Supplemental Figure S2. Prediction performance of the height (binary) trait under the TGBLUP and GBM under Data Set 1 for four proportions of the testing sets (0.2, 0.4, 0.6 and 0.8) for each environment in terms of the Kappa coefficient (Kappa). The whisker plots indicate the standard errors.

Supplemental Figure S3. Prediction performance in the categorical trait DTHD of TGBLUP and GBM under Data Set 1 for four proportions of the testing sets (0.2, 0.4, 0.6 and 0.8) for each environment in terms of the proportion of cases correctly classified (PCCC). The whisker plots indicate the standard errors.

Supplemental Figure S4. Prediction performance in the categorical trait DTHD of TGBLUP and GBM under Data Set 1 for four proportions of the testing sets (0.2, 0.4, 0.6 and 0.8) for each environment in terms of the Kappa coefficient (Kappa). The whisker plots indicate the standard errors.

Supplemental Figure S5. Prediction performance in the height (binary) trait under the TGBLUP and GBM under Data Set 2 for four proportions of the testing sets (0.2, 0.4, 0.6 and 0.8) for each environment in terms of the proportion of cases correctly classified (PCCC). The whisker plots indicate the standard errors.

Supplemental Figure S6. Prediction performance of the height (binary) trait under the TGBLUP and GBM under Data Set 2 for four proportions of the testing sets (0.2, 0.4, 0.6 and 0.8) for each environment in terms of the Kappa coefficient (Kappa). The whisker plots indicate the standard errors.

Supplemental Figure S7. Prediction performance in the categorical trait DTHD of TGBLUP and GBM under Data Set 2 for four proportions of the testing sets (0.2, 0.4, 0.6 and 0.8) for each environment in terms of the proportion of cases correctly classified (PCCC). The whisker plots indicate the standard errors.

Supplemental Figure S8. Prediction performance in the categorical trait DTHD of TGBLUP and GBM under Data Set 2 for four proportions of the testing sets (0.2, 0.4, 0.6 and 0.8) for each environment in terms of the Kappa coefficient (Kappa). The whisker plots indicate the standard errors.

Supplemental Figure S9. Data sets 1 and 2. Prediction performance of the height (binary) trait under the TGBLUP and GBM under four proportions of testing sets (0.2, 0.4, 0.6 and 0.8) across environments and both traits in terms of the proportion of cases correctly classified (PCCC) for (a) Data set 1 (across 5 environments), (b) Data set 2 (across 4 environments). The whisker plots indicate the standard errors.

Supplemental Figure S10. Data sets 1 and 2. Prediction performance of the height (binary) trait under the TGBLUP and GBM under four proportions of the testing sets (0.2, 0.4, 0.6 and 0.8) across environments and both traits in terms of the Kappa coefficient (Kappa) (a) Data set 1 (across 5 environments), (b) Data set 2. The whisker plots indicate the standard errors.

Supplemental Figure S11. Prediction performance for the categorical trait DTHD, of TGBLUP and GBM under four proportions of the testing set (0.2, 0.4, 0.6 and 0.8) across environments in terms of the proportion of cases correctly classified (PCCC). a) Data set 1, b) Data set 2, c) Data set 3, and d) Data set 4. The whisker plots indicate the standard errors.

Supplemental Figure S12. Prediction performance in the categorical trait DTHD of TGBLUP and GBM under four proportions of the testing set (0.2, 0.4, 0.6 and 0.8) across environments in terms of the Kappa coefficient (Kappa). a) Data set 1, b) Data set 2, c) Data set 3, and d) Data set 4. The whisker plots indicate the standard errors.
